# Experience of caregivers on the continuum of care and prevention of malnutrition among children with cholera in Ethiopia: a phenomenology study

**DOI:** 10.1186/s12889-024-18080-1

**Published:** 2024-02-26

**Authors:** Alemayehu Belay Alamneh, Kalkidan Hassen Abate, Ashagre Molla Assaye, Yeshambel Worku Demlie, Moti Edosa Guma, Tefera Belachew

**Affiliations:** 1 Public Health Emergency Management, Ethiopian Public Health Institute, Addis Ababa, P.O. Box: 1242, Ethiopia; 2 Institute of Health, Department of Human Nutrition & Dietetics, Jimma University, Jimma, Ethiopia; 3 College of Medicine and Health Sciences, Department of Nursing, Bahir Dar University Bahir Dar, Bahir Dar, Ethiopia

**Keywords:** Acute malnutrition, Caregivers, Children under fifteen years old, Cholera

## Abstract

**Introduction:**

Malnutrition is a public health problem in low- and middle-income countries among children. Although illnesses such as diarrhea are common immediate drivers of childhood malnutrition, their consequences could be averted through optimal sick child feeding and care to ensure the continuum of care. This study aimed to explore the lived experiences of mothers/caregivers on continuum of care to prevent malnutrition among children with cholera in Ethiopia.

**Methods:**

A phenomenology study design was applied to explore experiences of mothers/caregivers in the Bale and Guji zones of the Oromia region, southeast Ethiopia, from November to December 2022 using an unstructured interview guide. The saturation of ideas was used to stop the in-depth interview. Translated data were cleaned and imported into ATLAS.ti7 software for analysis. Using an open coding system, the data were coded into a meaningful context. Deeper immersion into data with repeated reading, creating themes, subthemes, and family/category were carried out. In coding and categorization, multiple coders were involved. The finding was presented using well-spoken verbatim/quotes as illustrations and in narratives.

**Results:**

In this qualitative study, ten participants were taken to explore their lived experience on the continuum of care for children with acute malnutrition and cholera. The study found that poverty, expensive cost of living, and poor utilization of diversified food were challenges. Moreover, health facilities did not provide any services to mothers whose child was admitted for malnutrition treatment. Children five years and above were excluded from both therapeutic food and screening for malnutrition program. Interruptions of supplies, low attention given to child feeding, inadequate knowledge, and lack of time to prepare diversified food were the main findings.

**Conclusion:**

Poverty, poor feeding habits, supplies interruption and non-inclusion of malnourished children five and above in screening for malnutrition and in the therapeutic feeding program is missed opportunities that lead to decreased early detection and treatment of malnutrition among children with cholera. Moreover, mothers/caregivers did not receive any service from health facilities when their child was admitted for treatment of malnutrition. This situation forces them to stop treatment before their child recovers from malnutrition, which has a negative impact on the continuum of care and prevention of malnutrition. Therefore, we strongly recommend strengthening emergency nutrition within the country’s health system and revising the food and nutrition policy to incorporate emergency nutrition, with a particular focus on children under the age of fifteen. Additionally, it is important that the study’s recommendations underscore the significance of a multi-sectoral approach that involves collaboration among the health sector, government agencies, and non-governmental organizations. Moreover, adaptive agricultural products be made easily accessible to the community which is crucial in effective preventing and reducing malnutrition in children in the study and similar settings.

## Background

Malnutrition is the main public health problem of low- and middle-income countries, mostly among young children. It contributes significantly to their morbidity and mortality and makes them susceptible to different diseases. It is driven by multiple factors, including poverty, dietary intake problems, communicable diseases, food insecurity, poor child and maternal health care, and inadequate hygiene and sanitation facilities [1].

Food insecurity and acute infections such as diarrheal disease result in wasting. Measurement of wasting or thinness is often used to assess the severity of an emergency, with severe wasting highly linked to child mortality [2].

Community-based management of severe acute malnutrition (CMAM) comprises weekly or biweekly outpatient therapeutic program follow-up for therapeutic food distribution. Distance to health facilities and high opportunity costs for caregivers can exemplify major barriers to access. Providing training and workshops to the community could increase awareness to enable them to recognize the danger signs and symptoms of health problems at home to reduce the frequency of outpatient visits for treatment and increase coverage and health-seeking behavior of caregivers, which can prevent malnutrition and reduce public health impacts [3].

Cholera is an acute intestinal infection caused by ingestion of water or food contaminated with Vibrio cholera that leads to acute watery diarrhea. It is the most communicable disease; unless treated promptly, it quickly leads to severe dehydration and death. To declare a cholera outbreak, only one confirmed case is enough. It remains a human being threat and public health problem in the world, particularly in the poorest and most vulnerable community in Africa [4–6].

Cholera and malnutrition are significant public health problems worldwide. Malnutrition and cholera are strongly interconnected such that malnourished people are more likely to develop cholera infections. The common practice of constraining food during recovery from acute diarrhea is incorrect during and after cholera infection, and it has been shown that early oral feeding during infantile diarrhea leads to better recovery. It has also been shown that early oral feeding throughout infantile diarrhea leads to better recovery. For a long time, malnutrition has been a problem in Africa and is now complicated by several factors, such as the impact of infectious diseases, limited food availability, poor food intake, loss of appetite, metabolic disorders, and restriction of food during recovery from cholera [7].

Community management of acute malnutrition was introduced as an innovative strategy and is now integrated into routine health service delivery in 70 countries [8]. The CMAM surge approach builds on CMAM programming to ‘make health systems more resilient over time by making them better able to cope with periodic peaks in demand for services for acute malnutrition without undermining the capacity and accountability of government health actors [8, 9].

International Food Policy Research Institute Report states that in most countries CMAM coverage is below 50% [11]. The absence of evidence regarding the interaction between infections and malnutrition in relation to the continuum of care after infection had been controlled which shows unable to establish a clear link between malnutrition and the subsequent care provided following an infection like cholera. Additionally, it is important to note that the current continuum of care process lacks the inclusion of children from all age categories in the outpatient therapeutic feeding program [12, 13]. Ethiopia 2016, Demographic and Health Survey reported that stunting, wasting and underweight are very high [14].

A study conducted in Jimma and southwest Ethiopia revealed that the recovery rate and defaulter rate were higher than the internationally acceptable standard ranges [11, 12].

A study done in Ethiopia in 2020 showed that low referral linkage affects the continuum of care and increases the burden of the disease, as only 16% of sick children were referred to the next health facility by health extension workers [12].

Even though children under five years old are highly exposed to malnutrition, all age categories of children are vulnerable, with a marked effect on the prevention of malnutrition. There is no evidence on the interplay between malnutrition and cholera among children older than five years. Limited evidence on children other than under five years of age will be addressed qualitatively. Hence, the aim of the study was to explore the lived experience of caregivers whose child had acute malnutrition with a history of cholera on the continuum of care and prevention of malnutrition.

The findings of this study will have critical importance in planning programs for improving the nutritional status of children older than five years with illness.

## Methods and materials

### Study area and period

The study was conducted in Bale and Guji Zones of the Oromia Region, southwest Ethiopia, which are high priority cholera hotspot areas from November to December 2022.

In this qualitative research, a phenomenology design was carried out.

### Source and study population

All mothers or caregivers with children under 15 years old in the study area were considered the source population whereas mothers/caregivers who had children less than 15 years old and whose child had been malnourished with a history of cholera infection were the study population.

### Sample size and sampling technique

The sampling and data collection process were determined by saturation of the ideas [17–21]. In this study, ten participants were included, and parents who had experience of malnourished children with history of cholera infection were selected using purposive sampling methods. The data collection tool comprised knowledge, perception, and understanding of caregivers on the continuum of care and prevention of malnutrition. Relatively, adequate sources of information of participants were used during data collection on lived experiences on the continuum of care, knowledge, and perceptions about malnutrition prevention.

### Data collection methods

The data were collected using an unstructured interview guide to explore the lived experiences of caregivers/parents’ perceptions, knowledge, practices, causes, and challenges regarding the continuum of care and the prevention of malnutrition. The tool used to collect data was prepared in English and translated into Amharic and Afaan Oromo using an independent translator and pretested to check their appropriateness to the local context. To obtain adequate information about their lived experiences on the continuum of care and prevention of malnutrition, an in-depth interview with study participants was carried out. Since the qualitative study needs an immersion process, the research team conducted data collection using mobile recorder technology with the permission of respondents, and all interviews were recorded in addition to taking notes.

During the face-to-face interview, appropriate safety and precautionary measures for COVID-19 were maintained, including the use of sanitizers, wearing masks by the interviewer and interviewee, keeping appropriate physical distance, and avoiding direct sneezing toward another person.

### Tool and interview guides

An unstructured interview guide with probes was used to explore caregivers’ perceptions, knowledge, practices, causes, and challenges as well as lived experiences on the continuum of care and prevention mechanisms to improve acute malnutrition in children. The interview guide has acute malnutrition and related topics, such as the perception and knowledge of caregivers about acute malnutrition, the importance of dietary diversity for children, service delivery in malnourished children, and challenges of providing diversified food to children. These main questions contained possible probes to remind their experience and follow-up questions to gain a comprehensive understanding of care givers on the continuum of care, awareness/knowledge, perceptions, and practice on the prevention of malnutrition.

### Maintaining trustworthiness

Quality assurance controlling mechanisms were employed throughout the entire research process to ensure trustworthiness.

The principal investigator and research team monitored the data collection process in addition to the supervisors. During data collection, the data were cleaned, and audio recordings were transcribed verbatim on the same day of data collection.

The verbatim transcriptions were translated into English for analysis. The translated data were read line by line repeatedly to understand the context and meanings used to increase immersion in the data.

### Data analysis process

The translated data were cleaned and imported to ATLAS.ti version seven for analysis. Using an open coding system, the data were coded into meaningful contexts. Deeper immersion into data with repeated reading, creating themes, subthemes, family/category and coding were used for data analysis. Multiple coders contributed to the coding and categorization of the data. The codes were combined into family/category, subthemes and themes. The findings were illustrated using direct respondents’ well-spoken verbatim/quotes as illustrations and summarized using narratives.

## Results

Overall, ten participants were taken to explore their lived experience on the continuum of care for children with acute malnutrition. Of them, three were females, in terms of age three of them were in the age group 30–39 years and two of the respondents were grade 12 and above (Table 1).

**Table 1:** Age category, educational and occupational status of respondents (*N* = 10)

Variable	Age category (in year)	Frequency
Age	20–29	3
	30–39	7
Sex	Male	7
	Female	3
Educational status	Read and write	4
	10–12 Grade	4
	> 12 Grade	2
Occupational status	Farmer	10

**Fig. 1:**
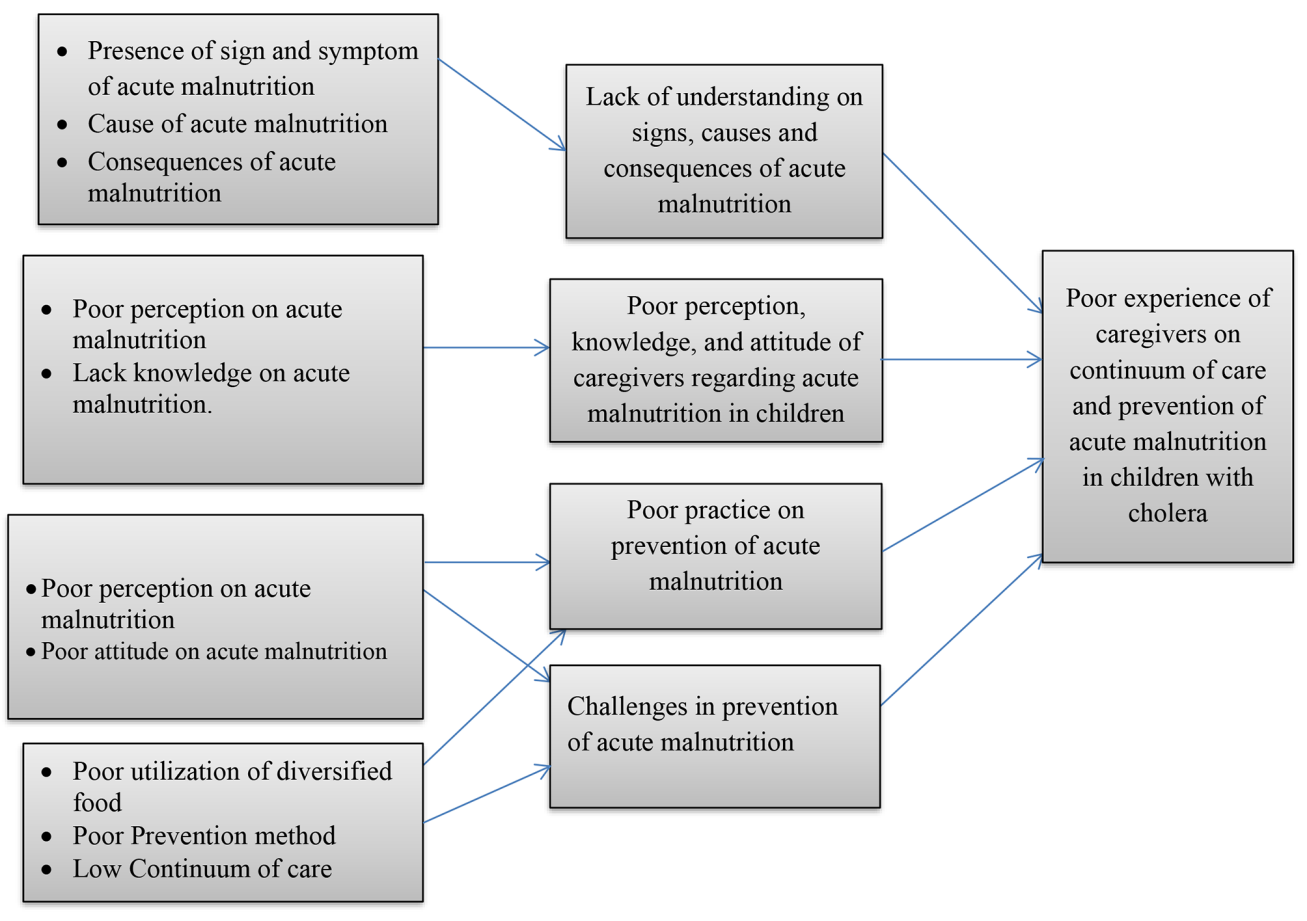
Linkage of thematic areas with the outcome

### Theme I: lack of understanding of the signs/symptoms, causes and consequences of acute malnutrition

#### Poor understanding of signs and symptoms of acute malnutrition

Some of the respondents did not know and explain the signs and symptoms of acute malnutrition in children, whereas other respondents described as follows: when children have malnutrition, they became thin, hungry, had reduced body weight, had defective mental growth, were stunted, and were exposed to different illnesses (28- and 35-year-old males).

Some respondents stated that when children became malnourished, they were predisposed to various risks, diseases or showed rashes on their body, wrinkles of skin or swollen bodies. Moreover, the participants uttered that the child would be weakened and unable to perform the tasks as well-nourished children.

*“…A malnourished person shows these symptoms: shrinking body, swollen stomach, thin leg when we pinch the skin not returned back, there will be mental retardation, and as experts told us that children who are malnourished their stomachs are not proportional to their bodies, and their necks are thin*.” (32-, 34- and 38-year-old male).

#### Poor understanding of causes and consequences of malnutrition

When asked about the causes of malnutrition, the common responses given were current expensive market conditions, poor standard of living, poor dietary practices, and behavioral factors such that people want to eat only one kind of food they need rather than having diversified food. They were hear voicing.

*“…There are causes of malnutrition like poor standard of living has its own effect, our eating behavior itself is a factor, and most people do not want to eat a kind of food differ from usual type, their need is only to eat the same types of food.”* (35 years old male).

Some respondents explained about the causes of malnutrition that poverty is the root cause, whereas lack of food is an immediate cause of malnutrition. On the other hand, there is a lack of knowledge about the consequences of malnutrition and poor feeding practices in society, which means that if children have enough food until their stomach is full, parents are not worried about the diversity of food made from different food sources, such as grains, fruits and animal products were identified causes. One of the respondents shared the following ideas:

*“…Poverty is the root cause of malnutrition, but lack of food and knowledge on consequences are the immediate causes. People are often told in our society what will happen to a child if his stomach is full. We heard food type should be from different kinds of grains or animal products and from fruits.” (35 years old male)*.

Some respondents stated lack of economy, lack of appetite, and poor traditional feeding practice that means poor attention is given to children and their food are the main causes. Unable to separate child food and their feeding time from adults’ also other problems; as a result, children are forced to eat adult food during feeding time, which shows that the food preference of children is ignored. Some of the mothers stated that it was very difficult to feed children different types of food because of poverty even and lack of knowledge on how to prepare diversified food even if supplies are available in the market; there was an accessibility problem in the area where they were living.

*“… Even if it (food items) exists in the zone, it will not reach the area where we are. Therefore, it is very difficult because poverty is hard. Even if grain is available, lack of knowledge on how to make it (prepare diversified food) also other problem.” (30 years old female)*.

Wonder full respondent explained that poverty was the major factor of malnutrition, lack of attention to the consequence of malnutrition, even though parents had different grains they did not prepare nutritious food from variety of grains so that their children suffered from malnutrition problems. In addition, the frequency of feeding and types of food given to children, which means that children eat a maximum of three times per day as adults even if they do not get as they feel hungry, are influencing factors.

Here, shared voice experience of respondent.

*“…Currently, it is known that there is no one who does not suffer from poverty and does not know the consequence of lack of nutrition or lack of attention on how to prepare nutritious food while having grains. Hence, there are people who have grain, and their children are suffering from malnutrition.” (38 years old male)*.

Some respondents viewed the cause of malnutrition as low attention given to child feeding and the attitude of selling important food ingredients like chicken, eggs, goats, sheep or expensive properties for closes or other equipment not for food. This shows lack of education and awareness of the family, showing that most of the parents in the community did not use diversified food even if they had no problem with income.

*“…It is difficult to provide variety and nutritious diet to children because of lack of income, lack of awareness and low attention is given to diversified foods. For example, when a family has chicken, eggs, goats or sheep, they want to go to the market and sell them to buy clothes or other equipment. This shows lack of education and awareness of the family.” (32 years old male)*.

### Theme II: poor perception, knowledge, and attitude of caregivers towards acute malnutrition

#### Perceptions and knowledge of acute malnutrition

Some of the respondents perceived malnutrition when children did not obtain diversified and adequate food, which led to poor dietary habits, lack of nutrients, improper body growth and exposure to different health problems. This is supported by the following idea of the respondent.

*“…To me malnutrition means not eating a variety of foods at meal times. Since our dietary practices are poor, most of us are not eating diversified or disease-fighting foods and suffer from nutrient deficiency.”* (35 years old male).

Some of the respondents perceived that malnutrition is not only getting enough amount of food in each meal that is prepared from one type ingredient such as “teff” or corn only but also the food should contain a variety of ingredients. A type of food item should change by different ingredients to make a variety of foods, and then eating a small portion of mixed food in each meal time will have nutrients. Similarly, other respondents perceived that malnutrition is not getting or eating a variety of nutritious and energy-giving foods.

*“…Nutritious food is not necessarily made from meat, eggs or other expensive ingredients but also it can make from available and cheap ingredients that can make diversified food like disease-fighting or something mixed of them.” (35* years old male).

Some of the respondents stated their perception about malnutrition as *follows:*

*“…Children who do not eat nutritious food will be exposed to diseases, so not eating nutritious food can reduce their mental and body growth.” “…If the child always eats the same type of food for breakfast, lunch and dinner, it can cause serious problems in his physical and mental development.” (28 years old female)*.

#### Unfavorable attitude towards acute malnutrition

Some of the respondents explained about the traditional influence of food and feeding practices; even though eating a variety of food is important and protects children from malnutrition, cultural influences do not allow eating a variety of foods in each meal. Poor child feeding practices, poor attitudes and attention are given to children. During mealtime, if children refuse to eat the food they dislike, they are forced to eat foods available in the house because food choice for children is not allowed.

*“…Most of the time people or children use the same types of food instead of eating different types of foods, which means preference of food is not allowed. For example, you cannot say that I do not like such kind of food. They are forced to eat only what is given to them. Therefore, due to these attitudinal problems, it is difficult to eat different types of foods.”* (35 years old male).

### Theme III: poor practice in the prevention of acute malnutrition

#### Low utilization of diversified food

Some respondents explained that many parents in the society have poor utilization of diversified food for many reasons, such as lack of awareness, ignorance, and low attention. Even if the root cause of malnutrition is poverty, lack of technical skill to prepare diversified food “mitin”, having another priority of the community, is responsible for the low utilization of diversified food.

Most of the respondents stated that even though diversified food is important for child development, such as mental alertness, body strength, willingness to work, and happiness, poor practice of diversified food utilization is a contributing factor for malnutrition. In addition, well-nourished children have proper growth, are able to understand things easily, and will have creative mind as whole they would have healthy life. However, this low practice prevents parents from giving attention to children and their feeding, which has a negative effect on the prevention of malnutrition.

“…*We know that when children get enough amounts of nutritious food, they will have a proper and healthy body as well as a proper working mind that can understand things easily, but children didn’t get such foods for so many reasons.” (*32 and 35 years old male).

#### Poor prevention method practice

Amazing respondent identified method of malnutrition prevention, as it is known that poverty is one of the causes of malnutrition acting upon it were important to prevent the malnutrition problems therefore, increase our wealth or income, increase the frequency of our diet by consulting health professionals or people who know more about nutrition, and pay attention to diversified food preparation and consumption, which are the main prevention mechanisms of malnutrition.

*“…Increase our wealth or income and increase frequency of feeding time by consulting health professional and increase our money for self-sufficiency. Eating too much does not indicate a balanced diet. Eating a variety of foods can prevent malnutrition.” (*35 years old male).

Some of the respondents’ explanations on the prevention of malnutrition were screening children for malnutrition, visiting health facilities for checking and receiving advice from health professionals about the preparation and utilization of diversified food, which were malnutrition prevention mechanisms. Moreover, poor traditional feeding practices should be improved, and special attention should be given to children and their food to include vegetables, fruit, and nutrients that can restore the health of wanting children and prevent malnutrition.

*“…when signs of malnutrition are shown on children, I will go to health facilities for treatment. On the other hand, health extension workers may come to our village for screening. At that time, all under-five children were screened for malnutrition, and advice was given on the prevention of malnutrition. I understand that if we prepare and feed diversified foods that contain vegetables, fruits, and nutrients to children, even if they have nutritional deficiencies, we can restore the health of deficient children by adjusting their diet.” (*38 years old male).

Most of the respondents explained three important prevention methods of malnutrition first, changing our poor dietary habits through feeding variety/nutritious food by assembling different ingredients such as fruits, vegetables, and eggs from nearby available areas. Second, malnutrition can be prevented or stopped by adjusting the feeding time of traditional food available at home with adequate amounts. Third, health professionals were consulted about diet and feeding practices or consulting people who had better knowledge of nutrition and diet.

*“…We can use many options to prevent or stop malnutrition, for example, we can use diversified food that is available from the surrounding areas. We can change our dietary habits, preparing and eating different foods from what we have at home. It is necessary to solve the problem by consulting health experts or by consulting people who can think and have better knowledge than me.” (*28 years old female).

>

#### Poor referral linkage and practice of the continuum of care

The respondent talked about the service continuity when signs of food deficiency were shown on children we had to go to the health post or health center for consultation service. Sometimes we have got consultation service by calling phone to experts or if we have an appointment goes to health facility with appointment card that was given from health facility (35 years old male).

Some respondents also explain that about the service given to malnourished children starting from getting advice and counseling from health professionals to recognizing physical change of the malnourished child, regular screening for malnutrition by health extension workers, and if children were screened as malnourished then go to health post for life saving mineral called “Plumpy Nut” that is given to malnourished children until the child recovers from malnutrition. If children do not recover, they are sent to a health center or hospital using referral paper for better treatment.

“…*The process is started from health extension workers’ screening children for malnutrition. At that time, if the child becomes malnourished, we will follow the procedure and take them if they are treated at the health post level. There are food treatments that are monitored by the health post and will continue until they are cured. If the child does not get better, they send us to the health center by referral system.*” (38 years old male).

Some of the respondents explained that when children finished their treatment from the health center, follow-up service would continue to health post as we as to the community until the children had well recovered. However, there is no checking/confirmation mechanism weather children had started the food treatment or not after they were referred to lower level for outpatient follow-up at the health post or the community unless parents asked about child health conditions when health extension workers come to their village for screening. One of the respondents stated,.

*“…of course, there is no way to confirm that referred patients have started follow up for dietary therapy, but sometimes health professionals may come to the villages for screening or vaccination, and advice was given at that time.”* (35 years old male.

Many of the respondents explained that there is no checking mechanism of referred patients to health posts or to the community. Sometimes confirmation is made using an appointment card whether children had started follow-up or not at a health post after being referred from health center. Otherwise, the presence of positive changes in the child’s body was checked. Generally, the services processes were started from the community from screening or went to the health post for follow-up. If children had complications or did not recover, they were referred to higher health care levels for further treatment. When they recovered, advice was given from health professionals and returned to the community.

“…*There are children on food treatments, monitored by the health post and will continue until they are cured. If the child does not get better, they send us to the health center by referral. In general, the process starts from family to the extension and to the higher level. As far as I know, there is a possibility that they go beyond the health center.”* (38 years old male).

Some of the respondents explained that even though the process of referral linkage started from the community to health post and to a higher level of health facility for further treatment and back to the community when they recovered, there are poor referral linkages among health facilities and the communities that cannot confirm malnourished patients have recovered or started following the treatment, which affects the continuum of care and prevention of malnutrition.

### Theme IV: challenges in the prevention of acute malnutrition

#### Health facility-related challenges

According to most respondents, there are challenges at health facilities when they sought care for sick children. Although there is health service given to sick children according to level of seriousness, there is no food service given to mothers/caregivers, which creates difficulty in staying in the facility to care for sick children. Such challenges make the mother/caregivers go home before the child recovers from their malnutrition. Furthermore, there was no shelter of space where the mothers could stay in making them suffer from cold at night and sunlight during the day. Previously, there were NGOs that provided food services, including coffee ceremonies, to the mothers/caregivers of sick children admitted to health facilities. The participants were heard voicing the following in trying to explain the state of affairs:

*“…There are problems at the health center, for example, if a child is sick and admitted, the mother does not get any food and shelter while caring for her child. She gets cold at night and gets sunlight during the day, so she has a lot of trouble. Previously, various NGOs used to provide food to mothers, even coffee.” (*28 and 34 years old male).

The other challenges explained were that children in the age group of five years and above were not included either in the screening or in the therapeutic program, leading to a missed opportunity for the treatment and prevention of malnutrition. They suffer from malnutrition because they do not get any nutritional support or treatment as uttered by a key and in-depth interview informant as”*…In addition, the program does not include children over 5 years old, which is a problem and not right.” (*34 years old male).

According to the respondents, in most health facilities, there were no proportional numbers of health professionals and equipment to diagnose and treat malnourished children, leading to suboptimal care (advice only) for mothers who travel from remote areas voiced by one of the key informants as follows:

*“…I do not say that the health facilities have enough supplies, sometimes when I look at our area, the number of patients and the numbers of health professionals are not balanced.” (*35 years old male).

#### Community-related challenges

According to the respondents, there are religious and traditional challenges leading to poor health-seeking behavior and low family planning utilization practices. This makes low birth spacing between consecutive children setting in an intergenerational cycle of malnutrition. The other challenge is the fact that the mothers leave others at home to care for the sick child in the health facility for the duration of admission, which drives the mother to return to home before the sick child completes the treatment. In addition, in the study area, mothers are more responsible for the families than males but with low decisional power. Therefore, such community challenges affect the prevention of malnutrition. One of the key informants stated the following to show the situation:

*“…There are also situations where mothers take children away from health facility without completing the treatment of the sick child. There is a lack of money and a lot of pressures on the women because like they left other children at home for giving care to the sick child in the health facility. If the father takes care of children, it would be nice; otherwise, it would be difficult to stay at the health facility until the sick child recovers.”* (26 years old female).

## Discussion

In this exploratory qualitative study, findings showed poor experience of caregivers on the continuum of care and prevention of malnutrition. Poor referral practice, excluding children five years and above from the screening and therapeutic program, lack of service for mothers whose children were sick at a health facility, and inadequate knowledge on the benefits of nutritious food were identified as drivers of child malnutrition during illness, such as cholera. Moreover, poverty, low accessibility of supplies, lack of knowledge, lack of time to prepare diversified food, and low attention given to the children’s diet were mentioned as challenges. Similarly, a study done in Burkina Faso and Mali showed that the integration of prevention services into treatment programs for acute malnutrition was omitted [22].

Even though most of the community members knew the consequences of malnutrition, they did not prepare and feed diversified food to their children, which is consistent with the report of a study from South Africa and Kenya [23, 24], which showed that mothers’ low practice of diversified food preparation and perception were the challenges to the prevention of malnutrition.

The other identified problems in the study were low income, expensive cost of living, inaccessibility, poor feeding habits of children, adequate knowledge on the benefits of nutritious food, and attitude of selling important food ingredients such as eggs or chicken, and poor dietary practices were important to prevent malnutrition. Furthermore, low attention was given to child feeding such that children had no separate food and time for feeding from adults, which is in accord with the reports of others in Ethiopia [25, 26].

The findings showed that there was no mechanism to check whether children referred to the health post for follow-up service had attended the outpatient therapeutic program. Improvement of children who were on treatment had been checked using appointment cards or physical changes to children’s bodies, which showed the loss to follow-up in the referral linkage of the continuum of care, in agreement with the report of a study in Bangladesh that showed that there was a weak referral system anywhere in the health facility [27].

It was observed that children in the age group of five years and above were included neither in the screening nor in the therapeutic program, leading to a missed opportunity for the treatment and prevention of malnutrition. This finding is similar to the reports of a study in Nigeria [28]. Even though children under five years of age were more exposed to malnutrition, acute malnutrition can develop even in children above five years in the context of illnesses such as cholera. Unless all children with acute illness, such as diarrheal disease, are included in screening and treatment of malnutrition, there will be missed opportunities in the prevention and treatment of malnutrition. The treatment supplies are also treated only for under-five malnourished children [29]. This implies the fact that there is need for screening any child with illness for malnutrition and giving the treatment accordingly to reduce the consequence of the illness. Similarly, a study done in Yemen showed that out of admitted patients for malnutrition treatment, 15% defaulted because of cultural, facility, lack of information and security problems [30]. The implication of this challenge not only affects the mothers whose children were on treatment but also has an effect on the program. The community knew that there were no services for caregivers of malnourished children, so they decided not to go to health facilities even if their child had become malnourished. Conversely, in Bangladesh, most NGOs work at the grassroots level to prevent such community problems [27].

In this study, inadequate continuing training for the community and health professionals was one of the identified problems. A study done in Bangladesh also reported that there were shortages of trained human resources in health workers in the community and health staff at health facilities for the prevention and management of SAM [27]. The study also identified inadequate supply or interruption of supplies as a problem, which is consistent with the report of a study from Bangladesh that identified supply problems such as pulpy nuts and milk [24, 28].

## Conclusions

The study findings have given a clue to policymakers, programmers, and other concerned bodies should to revise the protocol and other decisions that influence health service delivery to combat malnutrition.

Exploration of the caregiver’s experience showed that there was no doubt about the vulnerability of children with illnesses such as cholera and the high burden of malnutrition among sick children. On the other hand, children five years and above were excluded not only from the food therapeutic program but also from screening for malnutrition, which needs to be considered in the future.

Hence, attention needs to be given to all age categories of children who have high exposure to acute malnutrition, ensuring supply interruption and revision of the protocol for screening and treatment of malnutrition during emergency situations such as cholera epidemics.

Its implication not only affects the mothers whose children were on treatment but also had a negative effect on the malnutrition prevention program. The community knows that there are no services given at health facilities for mothers/caregivers of malnourished children. Therefore, unless health facilities give full service to caregivers of admitted malnourished patients, it could have a negative impact on the continuum of care and prevention of malnutrition.

Low attention given to diversified food preparation and feeding, poor knowledge of malnutrition consequences, poor referral linkage, and inadequate training about malnutrition prevention were challenges to the prevention of malnutrition. To alleviate these problems, sustainable training, workshops, and community conferences should be held to increase knowledge and community awareness that enable to bring the intended behavioral change to prevent nutrition-related problems.

In summary, to effectively combat the identified challenges, it is crucial to take specific actions. We strongly recommend strengthening emergency nutrition within the country’s health system and revising the food and nutrition policy to incorporate emergency nutrition, with a particular focus on children under the age of fifteen [31]. Additionally, it is important that the study’s recommendations underscore the significance of a multi-sectoral approach that involves collaboration among the health sector, government agencies, and non-governmental organizations. Moreover, poverty is found a significant challenge; addressing poverty and malnutrition are vital aspect in the study and similar settings. Hence we strongly recommend that adaptive agricultural products be made easily accessible to the community which is crucial in effective preventing and reducing malnutrition in children. By enhancing agricultural productivity, promoting sustainable farming practices, and empowering communities, it becomes possible to break the cycle of poverty and malnutrition, leading to improved health outcomes and overall well-being for children in the study and similar settings.

## Data Availability

The materials and dataset we used in this study are available upon reasonable request to the corresponding author and the approval of Ethiopian Public Health Institute or Jimma University.
